# Correlation and mechanism between cardiac magnetic resonance imaging and oral streptococcus count in patients with primary microvascular angina pectoris

**DOI:** 10.1097/MD.0000000000029060

**Published:** 2022-03-25

**Authors:** Qi Huang, Shi Sheng Wang, Rong Hua Luo

**Affiliations:** *Affiliated Hangzhou Chest Hospital, Zhejiang University School of Medicine (HangZhou Red Cross Hospital), Hangzhou, 208 Huancheng East Road, Zhejiang, China.*

**Keywords:** cardiovascular magnetic resonance, fibrinopeptide A, homocysteine, platelet alpha-granule membrane glycoprotein 140, primary microvascular angina, *Streptococcus sanguinis*, von Willebrand factor

## Abstract

**Background:**

Although primary microvascular angina (PMVA) can be diagnosed clinically, the etiology and pathophysiology of PMVA remain unclear. The effects of conventional clinical medications (aspirin, statins, and nitrates) are unsatisfactory, and PMVA can lead to serious cardiovascular events. The present study was designed to analyze the correlation between the load perfusion cardiovascular magnetic resonance imaging (CMR) results and the *Streptococcus sanguinis(S sanguinis)* count and the correlations between the *S sanguinis* count in oral cavity subgingival plaque and changes in the plasma levels of platelet alpha-granule membrane glycoprotein 140 (GMP-140), fibrinopeptide A (FPA), von Willebrand factor (vWF), and homocysteine (Hcy) in patients with PMVA after increased anti-infective treatment of the oral cavity. This study also discusses the pathogenesis of PMVA from this perspective. The differences in the *S sanguinis* count in oral cavity subgingival plaque and oral health status between healthy people and PMVA patients will be compared, and the correlation between the oral cavity health status and disease in PMVA patients will be analyzed.

**Methods:**

The present randomized controlled trial with a parallel control group will be conducted in 68 PMVA patients diagnosed by the in-patient cardiology department. The selected patients will be randomly divided into 2 groups, one receiving routine drug treatment and the other a combination of anti-infective treatments. The normal control group will comprise 30 healthy people with no infectious oral cavity disease matched by age and sex. We will conduct CMR, and the presence of *S sanguinis* in subgingival plaques will be used to determine the bacterial count in PMVA patients. Blood samples will also be collected to determine the levels of GMP-140, FPA, vWF, and Hcy. *S sanguinis* in the subgingival plaque of PMVA patients will be further analyzed after increasing the oral cavity anti-infective treatment; the resulting changes and their correlations with changes in GMP-140, FPA, vWF, and Hcy levels will be assessed. Additionally, the differences in the *S sanguinis* count and the oral cavity health status of oral cavity dental plaque between healthy people and PMVA patients will be determined, and the correlation between the oral cavity conditions and PMVA will be analyzed. The relationship between the perfusion CMR results and the oral cavity *S sanguinis* count of PMVA patients, and the potential pathogenesis, will be explored. We will use the SPSS19.0 statistical software package to analyze the data. The measurements will be expressed as means±standard deviation. Student *t* test will be used for intergroup comparisons, a relative number description will be used for the count data, and the chi-square test will be used for intergroup comparisons. Multivariate logistic regression will be performed to identify associations. A *P* value < .05 will be considered significant.

**Discussion:**

In this study, the correlation between the perfusion CMR results and the *S sanguinis* count in oral cavity subgingival plaque of PMVA patients will be analyzed. Changes in the levels of GMP-140, FPA, vWF, and Hcy of PMVA patients after receiving increased oral cavity anti-infective treatment will be explored, and the difference in the *S sanguinis* count in oral cavity subgingival plaque and the oral cavity health status between healthy people and PMVA patients will be compared.

**{2a}Trial registration:**

Chinese Clinical Trial Registry, (http://www.chictr.org.cn/showprojen.aspx?proj=45091)

## 1. Introduction

{6a}With the rapid development of evidence-based medicine, interventional cardiology, and imaging cardiology, coronary microvascular disease (CMVD) has received increasing attention. CMVD is a clinical syndrome displaying objective evidence of labored angina pectoris or myocardial ischemia due to structural changes and/or dysfunction of the anterior small coronary arteries and arterioles caused by various pathogenic factors.^[1-5]^ The coronary arteries include the subepicardial coronary artery, the anterior small arterioles, and the arterioles. The anterior small arterioles and arterioles constitute the coronary arterial microvasculature,^[6]^ which significantly affects myocardial blood perfusion when the subepicardial coronary arteries are not stenosed.^[7,8]^ They are the main resistance vascular beds of the coronary arteries and the site of myocardial metabolism.^[9-11]^ Damage to the anterior small arterioles and arterioles can lead to varying degrees of microvascular disease.^[2]^ The coronary arterial microvasculature cannot be visualized by coronary angiography, and 20% to 30% of patients undergoing coronary angiography due to chest pain have nonobstructive lesions.^[12]^ In such cases, the presence of CMVD must be considered.

Most of the conclusions regarding CMVD are limited to clinical experience and inference. There are no large-scale randomized clinical trials that have evaluated CMVD as the study objective, with changes in coronary arterial microvascular function as the endpoint.^[13]^ Therefore, research is needed to understand the etiology, pathophysiology, diagnosis, and treatment of CMVD and thereby improve the prevention and treatment of this disease and reduce the rate of cardiovascular events.

CMVD is divided into 4 types based on the possible etiology^[14-17]^: CMVD in the absence of myocardial diseases or obstructive coronary artery disease, CMVD with myocardial diseases, CMVD with obstructive coronary artery disease, and iatrogenic CMVD. CMVD without obstructive coronary artery disease is also known as primary microvascular angina (PMVA).

The results of treadmill exercise tests are usually positive in patients with typical clinical symptoms of angina pectoris,^[18,19]^ the results of electrocardiogram examination are myocardial ischemia, and the results of coronary angiography are generally normal.^[20]^ PMVA should be considered in patients with evidence of myocardial ischemia but whose coronary angiography results are generally normal and in patients with exclusive spastic coronary artery lesions, transient coronary artery thrombosis, cardiomyopathy, or other cardiovascular diseases.^[20]^ The etiology and pathophysiology of the disease are unclear but may be the result of multiple factors, including atherosclerosis, coronary microvascular endothelial dysfunction, the inflammatory response, oxidative stress, insulin resistance, and abnormal lipid metabolism. Moreover, vascular endothelial dysfunction is the initial mechanism of various vascular diseases.^[21]^ The vascular endothelium is a defensive barrier that transmits information and is involved in the synthesis and secretion of substances affecting vasomotor function. The inflammatory reaction promotes vascular endothelial dysfunction and vascular remodeling, which affect vasomotor function and lead to microvascular structural sclerosis and abnormal regulation of blood flow.

The symptoms of chest pain in patients with PMVA are clear, but the treatment effect of routine clinical medications (aspirin, statins, and nitrates) is unsatisfactory.^[21]^ This leads to repeated and excessive medical treatments, performance of various examination measures, and an increased psychological and economic burden on patients. PMVA can also progress to serious cardiovascular events, as the rates of major cardiovascular events and all-cause mortality in patients with PMVA are significantly higher than those of the control population, so early detection and effective treatment are important.^[22]^

The current invasive technique used to evaluate coronary microvascular function is coronary angiography, which evaluates perfusion of the visible epicardial coronary artery. When the epicardial coronary artery is not significantly narrowed, the blood flow speed reflects the level of microvascular resistance.^[23]^ The main noninvasive technique for evaluating coronary microvascular function is cardiovascular magnetic resonance (CMR),^[7]^ which is highly repeatable, has high tissue resolution, and uses nonionizing radiation. This “one-stop” scanning procedure can be used to assess cardiac morphology, function, myocardial perfusion and metabolism, myocardial microcirculation, and provide other information; thus, it has become the “gold standard” for the noninvasive assessment of cardiac structure and function.^[9]^

Perfusion CMR reflects the microcirculatory function of the myocardium via uptake of contrast medium during different metabolic periods of the myocardium. The change in signal intensity after intravenous injection of gadolinium contrast agent is used to assess microvascular obstruction.^[24]^ Gadolinium contrast is an extracellular contrast agent. After entering the blood, gadolinium quickly diffuses into tissue gaps through the capillary wall, but it cannot pass through walls with normal structure and function. A microcirculatory disorder is indicated by a slower increase in the signal strength, and thus a relatively low signal area, in the ischemic area compared with the adjacent cardiac muscle segment. As the perfusion time is extended, the contrast agent in the extracellular interstitial space is removed rapidly, and most of the contrast agent is discharged from the interstitial space when delayed scanning is performed. Therefore, normal myocardium does not show delayed enhancement, while myocardium associated with a microcirculatory disorder shows delayed enhancement. The characteristics of perfusion CMR suggest that visualization of the microcirculation may be the most promising technique for accurately evaluating microcirculatory disorders.

Infectious diseases in the oral cavity, such as coronary atherosclerotic heart disease, often require many public health resources and pose a serious risk on human health. However, although the harm, prevention, and treatment of coronary atherosclerotic heart disease have attracted widespread public attention, bacterial infectious diseases common in the oral cavity are often ignored. A large number of bacteria colonize the oral cavity under physiological conditions, but they are conditional pathogenic bacteria that have a certain effect on the oral cavity and overall health.^[25,26]^

*Streptococcus sanguinis(S sanguinis)*, which usually resides in the nasopharynx and oral cavity, is an important bacterium in the oral ecosystem.^[27-29]^ It adheres mostly to the surfaces of soft and hard oral tissues^[29]^ and is an important component of oral cavity dental plaque. *S sanguinis* readily enters the circulation through damaged periodontal tissue during oral hygiene and oral treatment procedures, resulting in bacteremia.^[27,30]^ In general, a small amount of bacteria is quickly cleared by phagocytic cells in the liver, spleen, and lymph nodes. However, when abundant bacteria enter the blood, or the immunity of the host is reduced, the uncleared *S sanguinis* reaches the heart and capillaries. The platelet aggregation-related antigens on the surface of *S sanguinis* promote platelet aggregation^[31]^; these surface antigens and other bacterial components also activate coagulation to form thrombi, which promote thrombosis and stimulate a systemic immune response.^[32]^ Numerous inflammatory mediators are released at high levels upon infection with *S sanguinis*,^[25,33,34]^ during which lipopolysaccharides located on the bacterial cell wall also enter the circulation.^[25]^ The inflammatory mediators and lipopolysaccharides act together to damage endothelial cells and promote smooth muscle proliferation and fat degeneration.^[26]^ Furthermore, platelet aggregation, activation of the coagulation system, and impaired vascular endothelial function all lead to the development of atherosclerosis and jointly promote the formation of PMVA.

Platelet alpha-granule membrane glycoprotein 140 (GMP-140) is localized in resting platelet alpha particles and the Weber corpuscles of endothelial cells. After cells are activated, GMP-140 is quickly released into the plasma, mediating the response of activated platelets and endothelial cells. GMP-140 also plays a central role in inflammation and embolism and is a specific molecular marker reflecting platelet activation. Fibrinopeptide A (FPA) is released during the conversion of fibrinogen to fibrin, which occurs via the hydrolysis of fibrinogen molecules by thrombin. FPA represents the comprehensive effect of procoagulant factors acting on various pathways in the body and the coagulation system and is a better indicator of coagulation system activation in patients. von Willebrand factor (vWF) is a polymer glycoprotein secreted by the vascular endothelial cells that play a key role in platelet aggregation by mediating adhesion between platelets and vascular endothelial cells. vWF is also a specific marker of vascular endothelial cell damage. Homocysteine (Hcy) is an intermediate product of methionine metabolism that reflects the metabolic status of the vascular endothelium and is an independent risk factor for cardiovascular diseases.

Attaching importance to oral cavity hygiene, receiving regular oral cavity examinations, and timely removal of oral cavity plaque are essential for preventing and reducing systemic diseases caused by oral cavity dental plaque. However, the correlations among oral cavity health status, the *S sanguinis* count in oral cavity subgingival plaque, and perfusion CMR results are unclear, and the mechanism of correlation between them is unknown. Therefore, we hypothesized that *S sanguinis* in the subgingival plaque of the oral cavity enters the circulation through damaged periodontal tissue, and platelet aggregationrelated antigens on the surface of *S sanguinis* induce platelet aggregation. Simultaneously, the antigens on *S sanguinis* activate the formation of coagulatory reactions and thrombosis. A large number of inflammatory mediators are released upon infection with *S sanguinis*, stimulating the systemic immune response, and lipopolysaccharides on the bacterial cell wall work together to damage vascular endothelial cells and promote vascular smooth muscle proliferation and fat degeneration. As a result, atherosclerosis develops, and the rate of PMVA increases.

### 1.1. Research objectives

*S sanguinis* infection is detected at a low rate in blood cultures, and the number of oral cavity dental plaques is a sensitive indicator of the quality of oral cavity hygiene. Therefore, this study will conduct CMR in PMVA patients; the *S sanguinis* count in subgingival plaques will be used to determine the bacterial count and the correlation between load perfusion CMR and the *S sanguinis* count in subgingival plaque in PMVA patients. Blood samples will be collected to detect the levels of GMP-140, FPA, vWF, and Hcy. After increasing the oral anti-infective treatment, *S sanguinis* in subgingival plaque in PMVA patients will be analyzed again, and the correlations between the *S sanguinis* count and changes in GMP-140, FPA, vWF, and Hcy levels will be assessed. The differences in the *S sanguinis* count and health status of oral cavity dental plaque between healthy people and PMVA patients will be determined, and the correlations between the oral cavity conditions and PMVA will be analyzed. The relationship between perfusion CMR and the oral cavity *S sanguinis* count in PMVA patients, along with the potential pathogenesis, will be explored.

## 2. Methods

### 2.1. Registration

**Figure 1.**
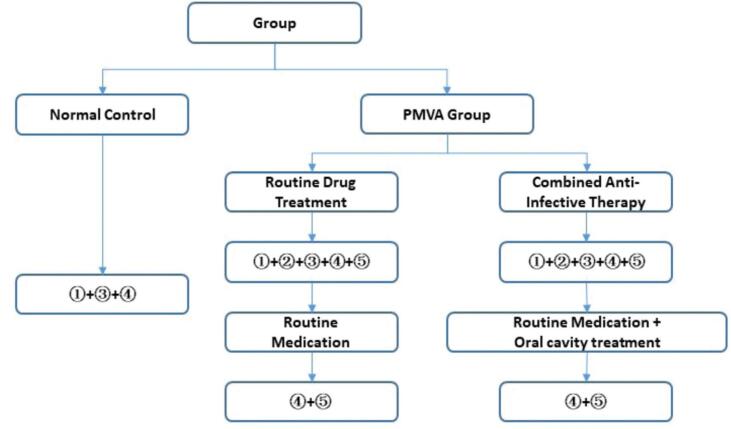
Notes: 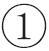 Record of general conditions. 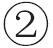 Cardiac examination. 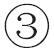 Oral cavity examination. 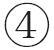 Collection, culture, identification, and counting of target bacterial specimens from subgingival plaques. 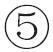 Measurement of plasma GMP-140, FPA, vWF, and Hcy levels. 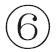 Routine medications: aspirin enteric-coated tablets 100mg qn, atorvastatin calcium tablets 20 mg qn, metoprolol succinate sustained-release tablets 47.5mg qd, and perindopril tablets 4mg qd, and nicorandil tablets 5 mg tid. 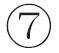 Oral cavity antiinfective treatments: supra-gingival cleansing, subgingival curettage for effective removal of plaque and calculus, and basic treatments such as root surface smoothing. Root surfaces were treated with hydrogen peroxide and povidone-iodine. Additionally, oral amoxicillin (0.5 g thrice daily) and metronidazole (0.2 thrice daily) were administered as antibiotic treatments for 7 days, combined with a gargle mouthwash (thrice daily for 1 mo) to combat Scutellaria. FPA = fibrinopeptide A, GMP-140 = platelet alpha-granule membrane glycoprotein 140, Hcy = homocysteine, vWF = von Willebrand factor.

{2a}This randomized, controlled, single-blind trial was registered before recruitment from the Chinese Clinical Trial Registry (http://www.chictr.org.cn/showprojen.aspx?proj=45091 No. CHiCTR2000030732). {2b}Does not appear in this manuscript but can be found in the trial registry record. We will conduct the trial based on the principles of the Declaration of Helsinki (2004 version). This study protocol was approved by the Ethics Committee of Hangzhou Red Cross Hospital before recruitment (approval number: 2020-2024). The study flowchart is shown in Figure 1.

{27}{29}Personal contact information will be accessible only to the research team members; all personal patient information will be protected. All information will be recorded using codes assigned to the patients (without mentioning patient names). {30} We do not anticipate any harm as a result of participation in this study. {31a}The results will be disseminated in journal publications. {25}The Ethics Committee of Hangzhou Red Cross Hospital will be informed of any modifications made to the protocol, and the new protocol will be uploaded to the CHiCTR.

### 2.2. Recruitment

{8}This randomized controlled trial including a parallel control group will be conducted on 68 patients in our cardiovascular department who agree to participate in the study and meet the inclusion criteria. The study protocol was prepared following the SPIRIT guidelines, which provide Standard Protocol Items. The SPIRIT checklist for the present study is provided in the Additional file.

{9}Affiliated Hangzhou Chest Hospital, Zhejiang University School of Medicine (HangZhou Red Cross Hospital) is located at 208 Huancheng East Road, Hangzhou. Hangzhou is the provincial capital of Zhejiang Province, China.

{15}{24}The first author will explain the research objectives and methods, including the purpose, processing, scheduling, and potential risks and benefits, to the participants before enrollment. {26a}The first author will obtain written consent from all patients willing to participate in the trial. {26b}No ancillary studies are planned. {21a}The ethics committee will monitor the entire procedure, including data collection. {21b}We do not plan to conduct an interim analysis.

### 2.3. Inclusion criteria

{10}The inclusion criteria are age 18 to 75 years and the following diagnostic criteria: typical exertional angina pectoris for at least 6 months, conventional 12-lead electrocardiogram recording and ST-segment offset measurement at 80 ms after the J-point or dynamic electrocardiographic monitoring showing at least 1 ST-segment ischemic descent ( ≥ 0.01 mm), positive treadmill exercise test (ST segment ischemic descent [ ≥ 0.01 mm]), normal echocardiographic left ventricular function and > 55% left ventricular ejection fraction, coronary artery angiography revealing normal or almost normal results (normal or irregular epicardial coronary artery wall, degree of stenosis < 20%), no spontaneous coronary artery spasm, and thrombolysis in myocardial infarction blood flow grade 1 to 2, and coronary microcirculation disorder confirmed by CMR.

### 2.4. Exclusion criteria

The exclusion criteria are organic heart disease, including coronary artery spasm, coronary artery bridge, primary cardiomyopathy, congenital heart disease, valvular heart disease, hypertrophic cardiomyopathy, pulmonary heart disease, or hypertensive heart disease; myocardial injury, including myocarditis and systemic combined myocardial injury; history of myocardial infarction, coronary intervention, or coronary artery bypass grafting; arrhythmia requiring drug control; complete left bundle branch block, degree II and III atrioventricular block, or sick sinus syndrome; severe congestive heart failure; malignant neoplasm; lung, esophageal, or aortic dissection or other diseases causing chest pain; severe liver or kidney dysfunction; acute or chronic inflammatory diseases or severe trauma; endocrine system diseases; anemia; peripheral vascular diseases; active biliary or hepatobiliary diseases; contraindication to resonance examination (magnetic field); inability to take medication; pregnant or lactating; penicillin allergy; and hypertension, hyperlipidemia, or diabetes.

### 2.5. Sample size estimate

Excel software will be used for data input. The chi-square test of multiple proportions was used to calculate the estimated sample size. The effect size of the chi-square test was 0.4, the significance level (alpha) was 0.05, the power of the test was 0.8, and the degrees of freedom were 2. A sample size of 68 was calculated based on these parameters, with 34 subjects in each group.

### 2.6. Study drug

{11a}Sixty-eight patients with PMVA will be selected and randomly divided into 2 groups, one receiving routine drug treatment and the other a combination of anti-infective treatments. The former group will be administered routine drug treatment (100mg aspirin enteric-coated tablets given orally once per night [Bayer Medical Health Co, Ltd, Chinese medicine quasi-word J20130078]; 20mg atorvastatin calcium tablets given orally once per night [Pfizer Pharmaceuticals Ltd Co, Chinese medicine quasi-word J20030048]; 47.5 mg metoprolol succinate sustained-release tablets given orally once daily [AstraZeneca, Chinese medicine quasi-word J20100098]; 4mg perindopril tablets given orally once daily [Servier Tianjin Pharmaceutical Co Ltd, Chinese medicine quasi-word H20034053]; and 5mg nicorandil tablets given orally thrice daily [Sino Foreign Pharmaceutical Co, Ltd, Chinese medicine quasi-word H20150023]).

### 2.7. Randomization and blinding

{16a-16c}A person not involved in the study will use a simple random technique to determine the allocation sequence with a 1:1:1 ratio. To ensure concealment of the allocations, this person will write the type of intervention based on the predetermined sequence and place it in an encoded opaque envelope. {17a}One of the coworkers will perform the interventional plan, and another coworker who knows nothing about the intervention will collect the data to ensure blinding. {17b}We do not predict any circumstances in which unblinding will be necessary.

The results will be gathered and the data imported into SPSS19.0 software. The researcher performing the data analysis will also be blinded to the type of intervention used for each group.

### 2.8. Interventions

The normal control group will comprise 30 healthy people with no oral cavity infectious diseases who will be matched with the treatment group by age and sex. Participants with diseases such as hypertension, hyperlipidemia, and diabetes will be excluded, and no selected participant will have taken antibiotics or hormones, used mouthwash containing antibiotics, or have undergone local periodontal treatment.

The following tests will be performed in the PMVA patients. Cardiac examinations will include routine electrocardiography, echocardiography, exercise load test, CMR, and coronary angiography. Target bacterial specimens in subgingival plaques will be collected, cultured, identified, and counted. An oral cavity examination will include determination of the periodontal index, including the periodontal pocket probing depth, gingival index, and plaque index. Six index teeth, nos. 16, 21, 24, 36, 41, and 44, will be assessed in each subject. Each index tooth has 6 test sites: 3 sites in the middle, near the middle, and far middle of both the lip (buccal) and tongue (palatal) sides. If the index tooth is missing, it will be replaced with another tooth in the same area. The periodontal index will be measured based on the average of the 6 index teeth, according to routine inspection of the periodontal index described in “Stomatological Preventive Medicine.” In addition, the number of teeth with periodontal disease, missing teeth, and dental caries will be recorded. General conditions will be recorded, including blood pressure, blood glucose and lipid levels, age, sex, education level (junior high school and below, high school, university, and above), monthly income ( ≤ 3000, 3000-10,000, ≥ 10,000yuan/mo), body mass index, smoking, alcohol consumption, number of tooth brushings per day, tooth brushing time, preference for salty or sweet food, and knowledge of the relationship between oral health and coronary heart disease, regular oral examinations, regular exercise habits, comorbidities, and family disease history.

The combination anti-infective treatment group will also receive oral cavity anti-infective treatments in addition to routine drug treatments, including supra-gingival cleansing, subgingival curettage to effectively remove plaque and calculus, and basic treatments, such as root surface smoothing. The root surfaces will be treated with hydrogen peroxide and povidone-iodine. Additionally, oral amoxicillin (0.5 g thrice daily) and metronidazole (0.2 thrice daily) will be administered as antibiotic treatments for 7 days, combined with a gargle mouthwash (thrice daily for 1 month) to combat Scutellaria.

{33}Coronary angiography will be performed before treatment, and plasma GMP-140, FPA, vWF, and Hcy levels will be measured simultaneously. Electrocardiography, dynamic electrocardiography, echocardiography, a treadmill exercise test, CMR, oral cavity examination, and collection, culture, identification, and counting of target bacterial specimens from subgingival plaques will be performed as well. Blood pressure, blood glucose and lipid levels, age, sex, education level, monthly income, body mass index, smoking, alcohol consumption, number of tooth brushings per day, tooth brushing time, preference for salty or sweet food, knowledge of the relationship between oral health and coronary heart disease, regular oral examinations, regular exercise habits, comorbidities, and family disease history will be recorded.

{11b}Participation in this study will not have a major impact on the daily life of the patients, and coronary angiography will be performed in our hospital. Coronary angiography is minimally invasive, and the relevant surgical risks will be specified preoperatively. Contraindication to a resonance examination is the only limitation of CMR. Blood analyses, electrocardiography, echocardiography, treadmill exercise tests, dynamic electrocardiography, oral cavity examinations, and collection, culture, identification, and counting of target bacterial specimens from subgingival plaques will all be safely performed. {6b}The drugs used in this study may have a low incidence of side effects, such as allergy, digestive discomfort, or abnormal liver and kidney function. These drugs have been widely used in clinical practice for many years and have been proven to be safe and effective. Regular inspections will be important, and treatments will be discontinued if serious complications occur.

{11c}The patients will be followed up 1 month later in the outpatient clinic, and the frequency of follow-up will be increased if necessary. The patients can contact the doctor if any side effects of the drugs are noted, in which case the study shall be terminated immediately and treatment started. {11d}The relevant concomitant care and interventions permitted during the trial will be performed.

*{13}*The cardiac examination, oral cavity examination, recording of general conditions, and collection, culture, identification, and counting of target bacterial specimens from subgingival plaques will be recorded in the PMVA and normal control groups before treatment. The PMVA group will additionally be examined for the *S sanguinis* count in subgingival plaque and plasma GMP-140, FPA, vWF, and Hcy levels before and 1 month after treatment.

#### 2.8.1. Coronary angiography.

Judkin's method will be used to evaluate the blood flow velocity of the epicardial coronary artery. The most commonly used evaluation index is the thrombolysis in myocardial infarction classification, the grades of which are as follows: 0, no blood flow past occlusion; 1, weak blood flow past the occlusion but no filling of the distal vascular bed; 2, blood flow reaching the distal vascular bed but slowed or delayed blood flow; 3, blood flow filling the distal vascular bed and normal blood flow.

#### 2.8.2. MR myocardial perfusion.

The first perfusion and late gadolinium enhancement (LGE) CMR scanning will be performed using the German Siemens 1.5T superconducting magnetic resonance scanner (Magnetom Avanto, Siemens) and gadolinium contrast agent.

#### 2.8.3. MR myocardial perfusion data analysis.

Segmental perfusion and LGE will be analyzed semiquantitatively. The segmental perfusion analysis is a visual analysis of each myocardial segment. According to the 17-segment method of the American Heart Association/American Heart Association, the wall motion of each segment is classified as grade 1 (normal movement), grade 2 (decreased movement), grade 3 (transportation), or grade 4 (dyskinesia). The LGE analysis uses a 4-point scale (0 = absolutely normal, 1 = may be normal, 2 = may be pathological, and 3 = pathological). Scores of 2 or 3 points are considered abnormal. The images will be blindly evaluated by 2 experienced radiologists, and the consensus used for analysis.

#### 2.8.4. The exercise load test.

The exercise treadmill test will be performed in the morning using the MAX-1 treadmill (Marquette Electronics Inc, Marquette, MI), with the sub-maximal load measured by electrocardiography and the Bruce scheme. A 12-lead electrocardiogram will be recorded before, during, and after exercise. A positive result is determined as the appearance of typical angina pectoris and level or oblique ischemic depression of the ST segment during and after exercise 80 ms after the J-point was > 1.0 mm and the depression of the ST segment lasted at least 1 minutes. The test will be terminated if one of the following conditions occur: typical angina attack, severe dizziness, sweating, severe asthma, pale skin, extreme fatigue, dyskinesia, and other symptoms; ST-segment depression ( > 0.2mV or T-wave inversion); severe arrhythmia (ventricular tachycardia, atrial fibrillation, multiple frequent ventricular premature beats, atrioventricular transmission); heart rate decrease to 25 beats/min or increase to > 85% of the maximum target heart rate; blood pressure increase to 220/110 mm Hg or systolic blood pressure decrease > 15 mm Hg; and subject asked to stop.

#### 2.8.5. Collection, culture, identification, and counting of target bacterial specimens in oral cavity gingival plaque.

1. Collection of oral cavity gingival plaque specimens. At least 2 teeth are natural in each area of the oral cavity examination and the collection time will be between 9:30 AM and 10:30 AM. After rinsing with clean water, the sampling site will be set as the mesial surface of the anterior and posterior teeth. Gingival plaque will be removed after drying the dental surface. The plaque will be scraped from the bottom of the periodontal bag using a sterile scraper. Subgingival plaques of approximately 2.5 mm in size will be transferred to a specimen bottle containing 0.9 mL transport medium and immediately inoculated and cultured.
2. Bacterial specimen culture. For the *S sanguinis* test, all specimen bottles will be thoroughly shaken using a vortex mixer for 2 to 3 minutes. The bacteria will be cultured in *S sanguinis* selective medium (NAYS-B) at 37°C for 48 hours in a 90% N_2_ and 10% CO2 environment. The API series of grampositive bacteria identification strips and VITEK2GP automatic identification instrument from Biomeria will be used for species analysis, identification, and colony counting.
3. Bacterial identification. The bacterial colonies of *S sanguinis* on NAYs-B selective medium will be 1to 1.5 mm in size, brownish-black, raised, and slightly rough on the surface.
4. Colony counting. The number of bacterial colonies will be counted.

#### 2.8.6. GMP-140 and FPA level measurements.

Fasting venous blood (5mL) will be added to a plastic test tube containing ethylenediamine tetraacetic acid anticoagulant, and the plasma will be separated by centrifugation at 3000r/min for 10 minutes and stored at -70°C. Enzyme-linked immunosorbent assay (ELISA) kits provided by Shanghai Xinfan Biotechnology Co, Ltd will be used to determine the levels of GMP-140 and FPA according to the operating procedures.

#### 2.8.7. vWF level measurement.

Venous blood (3mL) will be collected and placed in a plastic test tube containing a 1/10 volume ratio of 0.109mmol/L sodium citrate anticoagulant, separated by centrifugation at 3000r/min for 10minutes, and stored at -70°C. A vWF ELISA kit (ADI/Dellwin Co) was used to measure the level of vWF, according to the manufacturer's protocol.

#### 2.8.8. Hcy level measurement.

Venous blood (3mL) will be collected and placed in a clear glass tube to analyze hemorrhagic clearance and then stored at -70°C. The Hcy ELISA kit (ADI/Dellwin Co) will be used to measure the Hcy level according to the manufacturer's protocol.

## 3. Data collection and management

{18a}Sixty-eight patients with PMVA will be selected and divided randomly into 2 groups, one receiving routine drug treatment and the other a combination of oral cavity anti-infective treatments based on the routine drug treatment.

The following tests will be performed in the PMVA patients: cardiac examination; collection, culture, identification, and counting of target bacterial specimens from subgingival plaques; and oral cavity examination. General conditions will be recorded pretreatment. *S sanguinis* counts in subgingival plaque and plasma GMP-140, FPA, vWF, and Hcy levels will be determined before and 1 month after the treatments.

{18b}The patients will be contacted by telephone every 7 days to inquire about their current condition and treatment and any complications. If necessary, the patients can visit the hospital for further consultation to promote retention and complete the follow-up. The completed clinical outcome data of participants who discontinue or deviate from the interventional protocols will be collected

### 3.1. Evaluation

#### 3.1.1. Primary outcome.

1. We will determine the correlation between the perfusion CMR results and the *S sanguinis* count in oral cavity subgingival plaque in PMVA patients. The *S sanguinis* count may be greater in patients with more obvious microcirculatory disorders according to the CMR results.
2. We will determine the *S sanguinis* count in oral cavity subgingival plaque in PMVA patients of the 2 different treatment groups. The changes in GMP-140, FPA, vWF, and Hcy levels will be used to evaluate the changes in oral conditions, platelet aggregation activity, coagulation status, and vascular endothelial function. These indicators may improve more significantly in patients treated with the combination of anti-infective treatments compared with the routine drug treatment. By analyzing the correlations among these indicators, the pathogenesis of PMVA and new treatment options may be identified.

#### 3.1.2. Secondary outcome.

We will compare differences in the *S sanguinis* count in oral cavity subgingival plaque and oral health status between the normal control and PMVA groups. An abnormal oral cavity condition may be more frequent in PMVA patients.

#### 3.1.3. Adverse events.

{18b}The patients will be contacted by telephone every 7 days to inquire about their current condition and treatment, as well as whether they have any complications. {22}All adverse events will be documented during the intervention. If any adverse event occurs, they will be immediately reported, and the participant will receive the necessary treatment. We will analyze causality to determine the severity, and the relationships among the adverse events. Serious drug-induced adverse events will be reported to the ethics committee for discussion of whether the criteria should be modified. The completed clinical outcome data of participants who discontinue or deviate from the protocols will be collected.

#### 3.1.4. Patient and public involvement.

Neither the patients nor the public will be directly involved in the development of this study protocol. We will disseminate the results to the study participants via journal publications and research conferences.

#### 3.1.5. Statistical analysis.

{19}The SPSS19.0 statistical software package will be used to analyze the data. {20c}First, the nonnormally distributed variables will be subjected to the appropriate transformation procedure. {20a}The measurements will be expressed as the mean±standard deviation, and Student *t* test will be used for intergroup comparisons. {20b}A relative number description will be used for the count data, which will be compared between groups using the chi-square test. Multivariate logistic regression will be performed to identify associations. A *P* value < .05 will be considered significant.

## 4. Discussion

Coronary arterial microvessels play an important role in the myocardial blood supply, and their dysfunction causes varying degrees of microvascular disease. Typical clinical symptoms of angina pectoris, such as manifestations of myocardial ischemia on an electrocardiogram, provide evidence of myocardial ischemia. However, the results of coronary angiography are generally normal, and patients with exclusive spastic coronary artery lesions, transient coronary artery thrombosis, cardiomyopathy, or other cardiovascular diseases should be diagnosed with PMVA. The etiology and pathophysiology of PMVA remain unclear and may be the result of multiple factors, including atherosclerosis, coronary microvascular endothelial dysfunction, the inflammatory response, oxidative stress, insulin resistance, and abnormal lipid metabolism. However, the symptom of chest pain in patients with PMVA is clear, and the effects of conventional clinical medications (aspirin, statins, and nitrates) are unsatisfactory; thus, such patients can develop severe cardiovascular events. The incidence rates of major cardiovascular events and all-cause mortality are significantly higher in PMVA patients than in the control population, so early detection and effective treatment of PMVA are important.

The current clinically feasible technology that can directly evaluate coronary microvascular function is selective coronary angiography, and the preferred non-invasive technique is CMR. This “one-stop” scanning procedure provides data on cardiac morphology, function, myocardial perfusion and metabolism, and myocardial microcirculatory function. Thus, CMR has become the “gold standard” for noninvasive assessment of cardiac structure and function.

A large number of bacteria colonize the oral cavity under normal physiological conditions, but they are conditional pathogenic bacteria that have a particular impact on the oral cavity and overall health. *S sanguinis* is an important component of oral plaque. It readily enters the circulation through damaged periodontal tissue during oral hygiene and oral treatment actions. A platelet aggregation-associated antigen is expressed on the surface of *S sanguinis*. These antigens induce platelet aggregation and, along with other bacterial components, activate coagulation reactions to form thrombi, play an exogenous role in promoting thrombosis, and stimulate systemic immune responses. The body releases many inflammatory mediators when infected with *S sanguinis*, and lipopolysaccharides located on the bacterial cell wall also enter the blood during bacterial infection. Both work together to damage vascular endothelial cells and to promote the proliferation of vascular smooth muscle and fat degeneration. Furthermore, the aggregation of platelets, activation of the coagulation system, and impairment of vascular endothelial function promote the development of atherosclerosis and jointly promote the formation of PMVA.

This study will analyze the correlations of the *S sanguinis* count in oral cavity subgingival plaque with the CMR results and changes in plasma GMP-140, FPA, vWF, and Hcy levels in patients with PMVA after increased oral anti-infective treatment. We will also discuss the pathogenesis of PMVA. The differences in *S sanguinis* counts and oral health status in oral subgingival plaque between healthy people and PMVA patients, and the correlation between oral cavity health status and disease in PMVA patients, will be analyzed.

### 4.1. Trial status

{3}The protocol's version number and date are V1.0 and March 12, 2020, respectively. Recruitment will begin on January 4, 2022, and data collection will probably be completed on December 31, 2022. The data will be updated in the Chinese Clinical Trial Registry.

Table S1, Supplemental Digital Content, http://links.lww.com/MD2/A953

## Acknowledgments

Qi Huang extend the sincere gratitude to her lab employees, who will be putting considerable time and effort into laboratory evaluations. And also deeply indebted to all of the other tutors and teachers in Translation Studies for their direct and indirect help to her. Thanks for all friends who help her. Finally, She indebted to her whole family for their continuous support and encouragement.

## Author contributions

QH designed the study and prepared the protocol. QH and SSW performed the interventions, RHL carried out the data collection and completion of the questionnaires. All the authors scrutinized and confirmed the final protocol.

**Conceptualization:** Qi Huang.

**Data curation:** Rong Hua Luo.

**Formal analysis:** Qi Huang, Rong Hua Luo.

**Investigation:** Qi Huang, Rong Hua Luo.

**Methodology:** Qi Huang, Shi Sheng Wang.

**Project administration:** Qi Huang.

**Resources:** Qi Huang.

**Software:** Qi Huang.

**Supervision:** Qi Huang, Shi Sheng Wang.

**Writing** - **original draft:** Qi Huang.

**Writing** - **review & editing:** Qi Huang.

## Supplementary Material

SUPPLEMENTARY MATERIAL
